# Eosinophilic Granulomatosis With Polyangiitis Presenting as a Mononeuritis Multiplex Mimicking Guillain-Barré Syndrome

**DOI:** 10.7759/cureus.98202

**Published:** 2025-11-30

**Authors:** Mohamad Zikir Ismail, Fathin Hadi, Wan Syamimee Wan Ghazali, Bazli Md Yusoff

**Affiliations:** 1 Internal Medicine, Universiti Sains Malaysia, Kubang Kerian, MYS; 2 Radiology, Hospital Universiti Sains Malaysia, Kota Bharu, MYS

**Keywords:** eosinophilic granulomatosis with polyangiitis (egpa), guillain-barre syndrome (gbs), mononeuritis multiplex, p-anti-neutrophil cytoplasmic antibodies (p-anca), vasculitis

## Abstract

A 41-year-old Malay woman with a background of allergic rhinitis, nasal polyps, and eczema presented with bilateral foot numbness and weakness. Neurological examination revealed bilateral foot drop and sensory loss over the L5-S1 dermatomes. Nerve conduction studies showed bilateral peroneal and tibial axonal neuropathy. She was initially diagnosed with Guillain-Barré syndrome (GBS) and treated with intravenous immunoglobulin (IVIG), but showed no clinical improvement. Further evaluation revealed pansinusitis on CT imaging, vasculitic rashes, marked eosinophilia of 8.7 × 10⁹/L, and positive p-ANCA (1:160), leading to a revised diagnosis of eosinophilic granulomatosis with polyangiitis (EGPA) with mononeuritis multiplex. She responded well to corticosteroid therapy and methotrexate, with significant neurological improvement and no relapse observed at follow-up. This case highlights the diagnostic challenge of distinguishing EGPA from GBS in patients with acute neuropathy and highlights the importance of recognizing systemic features such as asthma, eosinophilia, and vasculitic signs to guide appropriate immunosuppressive treatment.

## Introduction

Mononeuritis multiplex is a common neurological manifestation of eosinophilic granulomatosis with polyangiitis (EGPA), also known as Churg-Strauss syndrome [1]. Meanwhile, the peripheral nervous system involvement observed in Guillain-Barré Syndrome (GBS), characterised by mixed axonal and demyelinating neuropathy affecting both sensory and motor nerves, can overlap with the neuropathic manifestations seen in EGPA [2]. In clinical practice, patients who initially present with acute bilateral limb weakness and areflexia may be misdiagnosed as having GBS. However, the subsequent appearance of systemic features such as asthma, sinusitis, eosinophilia, or ANCA positivity should prompt reconsideration of EGPA as the underlying cause [2,3]. Differentiating between EGPA and GBS, therefore, requires a dynamic diagnostic approach that integrates neurological, haematological, and systemic findings.

## Case presentation

**Figure 1:**
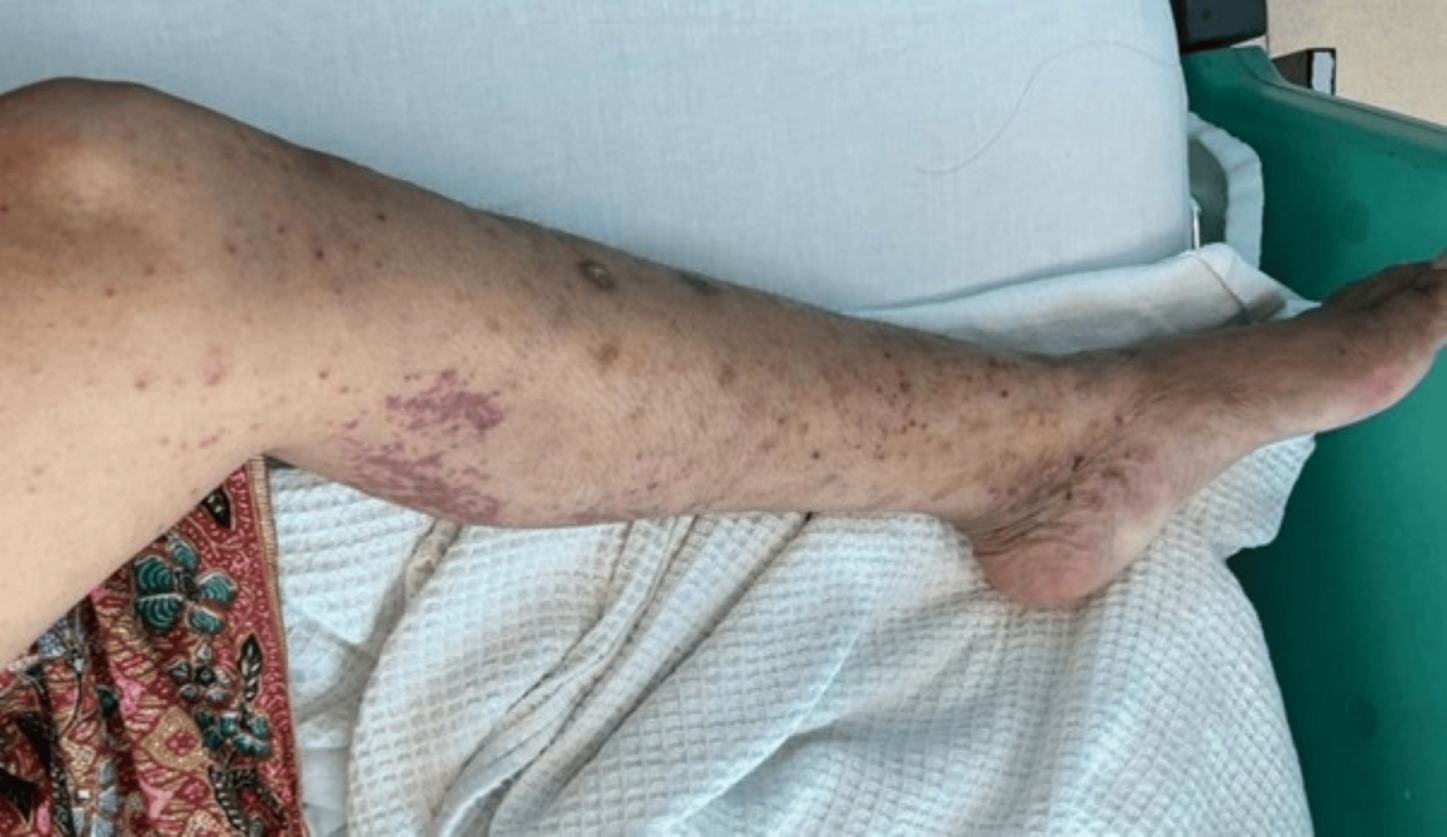
Vasculitic rashes on left lower limb (medially)

**Figure 2:**
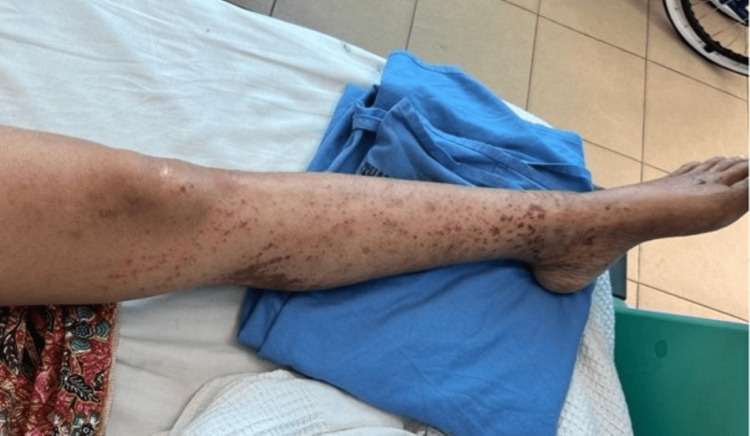
Vasculitis rashes on left lower limb (anteriorly)

**Figure 3:**
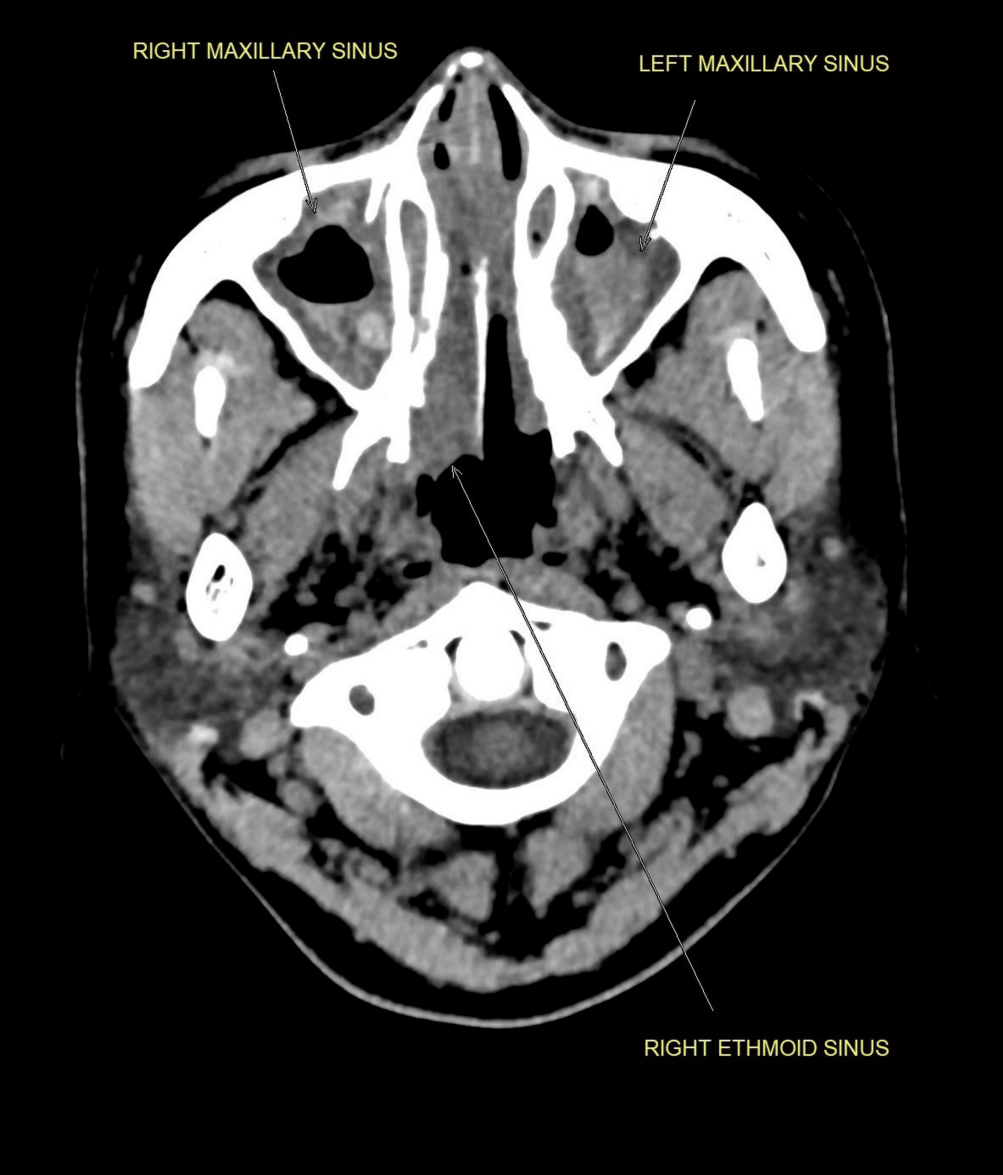
Non-contrast computed tomography of the head shows mixed iso-dense and hyperdense soft tissue material filling up the ethmoid and bilateral maxillary sinuses (yellow arrow)

**Figure 4:**
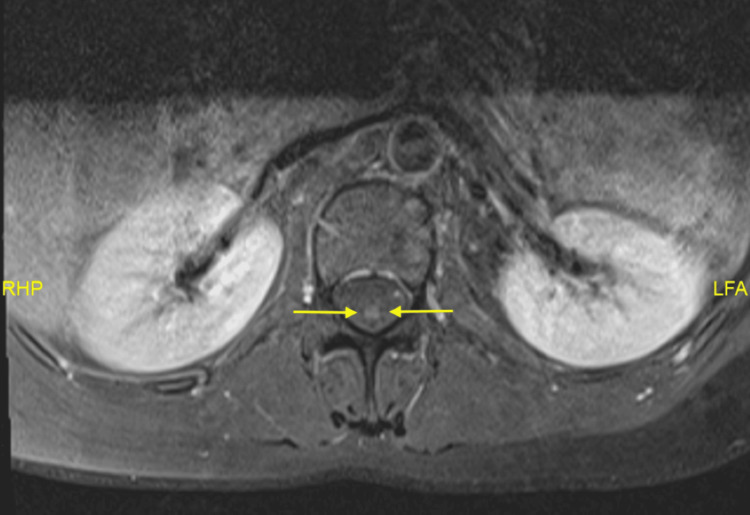
MRI lumbosacral with gadolinium (T1 sequence) shows enhancement of the central canal (yellow arrow)

We present the case of a 41-year-old Malay woman with a history of allergic rhinitis, nasal polyps, and chronic eczema. She presented with bilateral foot numbness and difficulty walking for two weeks. Neurological examination showed bilateral foot drop (3/5 strength) with impaired dorsiflexion, eversion, and inversion. Sensory loss was noted in the bilateral L5 and S1 dermatomes, and ankle reflexes were diminished, while knee reflexes remained intact. Gait analysis revealed a steppage gait. Further examination revealed maculopapular rashes on both lower limbs (Figures 1, 2). She was referred to dermatology for further evaluation, and the rashes were thought to represent vasculitis. A skin biopsy was planned, but the patient declined. A non-contrast CT scan of the brain showed no significant intracranial abnormalities. However, there was mixed iso-dense and hyperdense soft tissue material filling the ethmoid and bilateral maxillary sinuses (Figure 3), which may represent pansinusitis or chronic infection. An MRI of the lumbosacral spine revealed bilateral symmetrical peripheral nerve root enhancement, along with patchy enhancement of the dura and central canal (Figure 4). Nerve conduction studies indicated bilateral distal peroneal axonal neuropathy and tibial neuropathy (Table 1).

**Table 1:** Nerve conduction study (NCS) results NCS showed bilateral distal peroneal axonal neuropathy with reduced motor amplitudes and slowed conduction velocities, more severe on the right. Tibial nerves demonstrated additional axonal involvement. Sural sensory responses were preserved bilaterally, while superficial peroneal sensory responses were absent. Median and ulnar motor and sensory studies were within normal limits. Overall, the pattern was asymmetric and predominantly axonal, consistent with mononeuritis multiplex in eosinophilic granulomatosis with polyangiitis (EGPA), rather than a demyelinating process such as Guillain-Barré syndrome.

Nerve/Site	Latency (ms)	Amplitude	Conduction Velocity	Normal Amplitude (mV)	Normal Velocity (m/s)
Peroneal motor – right (popliteal segment)	3.6	2.3 mV	100 m/s	2–6 mV	≥50 m/s (approx for lower limb), ≥55 m/s (upper limb)
Peroneal motor – left (popliteal segment)	4.2	1.1 mV	59 m/s	2–6 mV	≥50 m/s (approx for lower limb), ≥55 m/s (upper limb)
Tibial motor – right (popliteal segment)	12.6	0.1 mV	42 m/s	4–20 mV	≥50 m/s (approx for lower limb), ≥55 m/s (upper limb)
Tibial motor – left (popliteal segment)	11.9	0.2 mV	51 m/s	4–20 mV	≥50 m/s (approx for lower limb), ≥55 m/s (upper limb)
Median motor – right	3.2	13.0 mV	63 m/s	4–15 mV	≥50 m/s (approx for lower limb), ≥55 m/s (upper limb)
Ulnar motor – right	3.3	6.8 mV	63 m/s	6–12 mV	≥50 m/s (approx for lower limb), ≥55 m/s (upper limb)
Sural sensory – right	2.1	14 µV	61 m/s	≥6 µV (sensory)	≥50 m/s (approx for lower limb), ≥55 m/s (upper limb)
Sural sensory – left	1.9	8 µV	68 m/s	≥6 µV (sensory)	≥50 m/s (approx for lower limb), ≥55 m/s (upper limb)
Superficial peroneal sensory – R/L	—	0 µV	—	≥6 µV (sensory)	≥50 m/s (approx for lower limb), ≥55 m/s (upper limb)

A diagnosis of GBS was made, and the patient began a five-day course of intravenous immunoglobulin (IVIG). After completing the IVIG course, her symptoms showed partial improvement, and she was allowed to be discharged.

Six weeks later, she was readmitted, complaining of sudden-onset numbness in her right hand. Neurological examination revealed sensory loss in the ulnar area of the right hand. Further tests revealed a positive p-anti-neutrophil cytoplasmic antibody (p-ANCA) result with a titre of 1:160. The complete blood count showed leukocytosis with an eosinophil count of 8.7 × 10⁹/L (45.8%) (Table 2). Antinuclear antibody testing was negative, and viral screening for hepatitis B and C was non-reactive.

**Table 2:** Laboratory result ANCA, antineutrophil cytoplasmic antibody; BUN, blood urea nitrogen; CRP, c-reactive protein; ESR, erythrocyte sedimentation rate; GFR, glomerular filtration rate.

Parameter	Value	Reference Range
WBC	18.99 × 10⁹/L	3.8–10.6 × 10⁹/L
Neutrophils (absolute)	7.9 × 10⁹/L	1.80–7.70 × 10⁹/L
Lymphocytes (absolute)	1.82 × 10⁹/L	1.10–4.00 × 10⁹/L
Eosinophils (absolute)	8.7 × 10⁹/L	0.00–0.70 × 10⁹/L
ESR	81 mm/hour	0–10 mm/hour
CRP	63.9 mg/L	< 5 mg/L
BUN	3.1 mmol/L	2.76-8.07 mmol/L
Creatinine	43 μmol/L	44-80 μmol/L
GFR (CKD-EPI)	122	
p-ANCA	Positive (1:160)	<1:20
c-ANCA	Negative	<1:20

Upon further inquiry, she had a history of frequent nebuliser use in 2023 and 2024 due to bronchospasm. Given the presentation of mononeuritis multiplex, vasculitic rashes, allergic rhinitis with nasal polyps, asthma, hypereosinophilia, pansinusitis on CT, and positive p-ANCA, the final diagnosis was EGPA with mononeuritis multiplex. She was started on high-dose prednisolone with a tapering regimen. During follow-up at the rheumatology clinic, she was started on weekly methotrexate 10 mg. After three months, she showed marked neurological improvement, with resolution of sensory deficits and partial restoration of motor function. No signs of disease relapse were observed, and corticosteroids were successfully tapered (Table 3).

**Table 3:** Timeline of the patient’s clinical presentation, investigations, and management

Time	Key Events	Findings/Actions
Week 0	Onset of symptoms	Bilateral foot numbness and progressive difficulty walking for two weeks
Week 2 (first admission)	Neurological findings	Bilateral foot drop (3/5), impaired dorsiflexion, eversion, inversion; sensory loss in L5–S1 dermatomes; diminished ankle reflexes; knee reflexes intact; steppage gait
	Cutaneous and dermatologic findings	Maculopapular rashes on both lower limbs (Figures 1, 2); dermatology consulted: suspected vasculitis; skin biopsy planned but declined
	Imaging	CT brain: no intracranial abnormality; pansinusitis changes (Figure 3)
	MRI lumbosacral spine	Bilateral symmetrical peripheral nerve root enhancement with patchy dural and central canal enhancement (Figure 4)
	Nerve conduction study	Bilateral distal peroneal and tibial axonal neuropathy
	Working diagnosis and management	Guillain-Barré syndrome (GBS) diagnosed; treated with IVIG for 5 days → partial improvement → discharged
Week 8 (readmission)	New neurological symptom	Sudden-onset numbness in the right hand (ulnar distribution)
	Laboratory findings	Leukocytosis with marked eosinophilia (8.7 × 10⁹/L; 45.8%), positive p-ANCA (1:160), negative ANA, non-reactive hepatitis B/C
	Systemic features identified	History of bronchospasm and nebulizer use; CT showing pansinusitis
Week 9	Revised diagnosis	Eosinophilic granulomatosis with polyangiitis (EGPA) with mononeuritis multiplex confirmed based on systemic, hematologic, and neurologic findings
Week 9 onward (follow-up)	Treatment and response	High-dose prednisolone (tapered) and methotrexate 10 mg weekly → marked neurological improvement after 3 months: no relapse on follow-up

## Discussion

EGPA and GBS are distinct disorders with overlapping clinical and pathological features, leading to diagnostic and therapeutic considerations. EGPA, formerly known as Churg-Strauss syndrome, is a systemic vasculitis characterised by eosinophil-rich granulomatous inflammation that affects multiple organs, most commonly the lungs, skin, and peripheral nerves [4]. In contrast, GBS is an acute, immune-mediated polyneuropathy characterised by progressive muscle weakness and sensory disturbances, often triggered by preceding infections, with subtypes including acute inflammatory demyelinating polyneuropathy and acute motor axonal neuropathy [5]. Both EGPA and GBS involve the peripheral nervous system, manifesting as neuropathy, which can lead to diagnostic challenges. Thus, differentiating between EGPA and GBS is crucial for appropriate management, as the treatment strategies differ significantly.

Although GBS was initially suspected based on the patient’s subacute weakness and bilateral sensorimotor involvement observed through the clinical, radiological, and neurophysiological tests, the evolving clinical picture suggested an alternative aetiology. The presence of multiplex mononeuritis, eosinophilia, positive p-ANCA, and systemic features, including asthma, sinusitis, and vasculitic rashes, strongly favoured EGPA-associated neuropathy over GBS.

Our patient initially presented with neurological symptoms mimicking GBS. However, persistent progression despite IVIG, eosinophilia, and p-ANCA positivity helped differentiate EGPA. This highlights the need for clinicians to consider ANCA vasculitis in GBS-like presentations, particularly in patients with allergic or eosinophilic histories.

The 2022 American College of Rheumatology and European Alliance of Associations for Rheumatology Classification Criteria for EGPA outline specific criteria for diagnosing EGPA, with each criterion assigned a particular weight. These include a maximum eosinophil count ≥1 × 10⁹/L (+5), obstructive airway disease (+3), nasal polyps (+3), cytoplasmic ANCA or anti-proteinase 3-ANCA positivity (-3), extravascular eosinophilic predominant inflammation (+2), mononeuritis multiplex or motor neuropathy not due to radiculopathy (+1), and haematuria (-1). After ruling out other conditions that mimic vasculitis, a patient with small- or medium-vessel vasculitis can be classified as having EGPA if their cumulative score is ≥6 points [6]. This system demonstrates high sensitivity (85%) and specificity (99%) for identifying EGPA patients. In our case, the patient scored 9 points, strongly indicating EGPA. While highly recommended, biopsies are not always practical and often yield non-specific findings [7]. If the peripheral nerves are involved and diagnostic ambiguity continues, a nerve biopsy might be an option. The nerve biopsy would show vasculitis, eosinophilic infiltration, and granuloma formation.

The mainstay of treatment for EGPA consists of corticosteroids and immunosuppressive drugs to control eosinophilic inflammation and vasculitis. On the other hand, GBS is usually treated with intravenous immunoglobulin or plasma exchange to eliminate or neutralise the harmful autoantibodies. While both conditions are immune-mediated, they involve different targets and pathways, requiring distinct treatment strategies. According to the 2022 EULAR recommendations, for induction of remission in patients with new or relapsing EGPA with organ- or life-threatening disease, a combination of glucocorticoids and either rituximab (RTX) or cyclophosphamide (CYC) is proposed. For non-organ- or non-life-threatening EGPA, glucocorticoids combined with RTX are recommended, with methotrexate (MTX) or mycophenolate mofetil (MMF) as alternative options to RTX. In our case, the patient was started on MTX [3].

The prognosis of EGPA-related neuropathy varies depending on the extent of nerve damage and the timing of treatment initiation. Studies indicate that with early diagnosis and appropriate immunosuppressive therapy, significant neurological recovery can be achieved in most patients [8]. However, delayed treatment or severe nerve involvement may lead to persistent deficits, including residual weakness, sensory loss, and chronic pain. In some cases, long-term immunosuppression is required to prevent disease relapse. A retrospective study on EGPA neuropathy found that up to 70% of patients showed partial or full recovery of nerve function after immunosuppressive therapy, although some required ongoing steroid-sparing agents such as methotrexate or azathioprine to maintain remission [1]. In contrast, GBS has a more predictable recovery pattern, with most patients regaining function within 6-12 months, although some experience residual fatigue, weakness, or autonomic dysfunction [9]. In this patient, the early introduction of corticosteroids and methotrexate led to neurological improvement, but long-term follow-up is essential to monitor for relapse, steroid dependence, or progression to systemic vasculitis.

This case highlights the importance of differentiating EGPA-associated neuropathy from GBS, given the differing pathophysiology and treatment approaches. The patient responded well to corticosteroids and methotrexate, emphasising the importance of early recognition and intervention to prevent long-term neurological sequelae (Table 4).

**Table 4:** Comparison of clinical, laboratory, and radiologic features observed in the present case, contrasting findings that supported EGPA with those initially suggestive of GBS

Feature	Eosinophilic Granulomatosis With Polyangiitis (EGPA)	Guillain-Barré Syndrome (GBS)
Clinical onset	Subacute onset over 2 weeks with progressive bilateral foot numbness and weakness	Usually acute, rapidly progressive (within days) ascending weakness
Symptom distribution	Asymmetric involvement (bilateral foot drop → later right hand numbness) consistent with mononeuritis multiplex	Symmetric ascending weakness and sensory loss
Reflexes	Diminished ankle reflexes with preserved knee reflexes	Global areflexia or hyporeflexia
Systemic features	Asthma, allergic rhinitis, nasal polyps, pansinusitis, and vasculitic rash on lower limbs	Typically absent; systemic involvement uncommon
Laboratory findings	Marked eosinophilia (8.7×10⁹/L, 45.8%), leukocytosis, positive p-ANCA (1:160)	Normal eosinophil count; may show mild albuminocytologic dissociation in CSF
Neurophysiology	Bilateral distal peroneal and tibial axonal neuropathy (asymmetric, motor-predominant)	Demyelinating or mixed axonal-demyelinating pattern, usually symmetric
Radiology (MRI/CT)	MRI lumbosacral spine: nerve root enhancement; CT sinuses: pansinusitis	MRI spine typically normal
Treatment response	Partial improvement after IVIG, but full response only after corticosteroids and methotrexate	Good response expected after IVIG or plasma exchange
Final diagnosis	EGPA with mononeuritis multiplex	GBS (initial working diagnosis)

## Conclusions

Mononeuritis multiplex is the most typical manifestation of vasculitic neuropathy in EGPA, but it can also resemble GBS, particularly when presenting with acute or subacute limb weakness. Nerve root enhancement on MRI may occur in both conditions; however, the asymmetric and patchy pattern often favours a vasculitic aetiology, whereas GBS more commonly demonstrates diffuse and symmetric enhancement. Although CSF analysis was not performed in this case, albuminocytologic dissociation would have supported GBS, while its absence would not exclude EGPA. Similarly, electromyography/nerve conduction velocity findings demonstrated axonal involvement of the peroneal and tibial nerves, which, although not specific, aligned more with mononeuritis multiplex than with the typical early demyelinating features expected in GBS. Clinical localisation of weak dorsiflexion, eversion, inversion, and sensory deficits in L5-S1 dermatomes further supported multifocal peripheral nerve involvement consistent with EGPA rather than the symmetric pattern classically seen in GBS.

The presence of systemic features such as asthma, sinus disease, eosinophilia, and vasculitic skin lesions should prompt reconsideration of EGPA, especially when there is poor or no response to IVIG. While partial recovery in demyelinating GBS may begin within the first month of treatment, full recovery usually evolves more slowly over 6-12 months, in contrast to the variable course of vasculitic neuropathy. Early recognition of these distinguishing clinical, electrophysiological, and imaging findings, together with prompt initiation of immunosuppressive therapy, is essential to prevent further neurological deterioration and improve functional outcomes. This case underscores the importance of a thorough clinical assessment and a multidisciplinary approach in atypical neuropathy presentations to ensure accurate diagnosis and timely management.

## References

[1] Cho HJ, Yune S, Seok JM (2017). Clinical characteristics and treatment response of peripheral neuropathy in the presence of eosinophilic granulomatosis with polyangiitis (Churg-Strauss syndrome): experience at a single tertiary center. J Clin Neurol.

[2] Khandelwal D, Singh A, Meena DS (2021). Eosinophilic granulomatosis with polyangiitis imitating Guillain-Barré syndrome: a case report. Egypt J Neurol Psychiatry Neurosurg.

[3] Quan MV, Frankel SK, Maleki-Fischbach M, Tan LD (2018). A rare case report of polyangiitis overlap syndrome: granulomatosis with polyangiitis and eosinophilic granulomatosis with polyangiitis. BMC Pulm Med.

[4] Vaglio A, Buzio C, Zwerina J (2013). Eosinophilic granulomatosis with polyangiitis (Churg-Strauss): state of the art. Allergy.

[5] Leonhard SE, Mandarakas MR, Gondim FAA (2019). Diagnosis and management of Guillain-Barré syndrome in ten steps. Nat Rev Neurol.

[6] Grayson PC, Ponte C, Suppiah R (2022). 2022 American College of Rheumatology/European Alliance of Associations for Rheumatology classification criteria for eosinophilic granulomatosis with polyangiitis. Arthritis Rheumatol.

[7] Hellmich B, Sanchez-Alamo B, Schirmer JH (2024). EULAR recommendations for the management of ANCA-associated vasculitis: 2022 update. Ann Rheum Dis.

[8] Chakraborty RK, Aeddula NR (2023). Eosinophilic granulomatosis with polyangiitis (Churg-Strauss syndrome). StatPearls [Internet].

[9] Khan F, Ng L (2009). Guillain-Barré syndrome: an update in rehabilitation. Int J Ther Rehabil.

